# Supplementary material for: Heuristics guide the implementation of social preferences in one-shot Prisoner's Dilemma experiments
Source: Sci Rep. 2014 Oct 28;4:6790. doi: 10.1038/srep06790 (PMC4210943; doi:10.1038/srep06790)
Supplement: Supplementary Information [file srep06790-s1.pdf]

Supplementary Information

for

Heuristics guide the implementation of social preferences in one-shot Prisoner's Dilemma experiments

Valerio Capraro, Jillian J. Jordan, David G. Rand

Welcome to this HIT. This HIT has two parts. We will tell you about the second part after you have completed the first one.

For your participation in this HIT, you receive 30 cents. You also can earn a bonus as described on the following pages.

How much of a bonus depends on your own decisions and also on the decision of an anonymous other MTurk participant with whom you are paired.

You will be told about the outcome of all parts of the HIT at the time your bonus is paid.

Supplementary Figure S1. Introductory screen.

This is the first part of the HIT.

You are together with another, anonymous participant. How much money you earn depends on your own choice, and on the choice of the other participant.

You are given 10 additional cents and you have to decide how much, if any, to transfer to the other participant. The amount of money you decide to transfer will be multiplied by 10 and earned by the other participant.

The other participant will be given the same choice.

So, for instance, if you both transfer everything, you both get 1 dollar and are better off than if you both keep everything. If you transfer everything and the other person keeps everything, then you earn nothing. If you keep everything and the other participant transfers everything, then you get 1 dollar and 10 cents.

The other person is REAL and will really make a decision. Once you have each made your decision, neither of you will ever be able to affect each others' bonuses in later parts of the HIT.

Now we will ask you several questions to make sure that you understand how the payoffs are determined.

YOU MUST ANSWER ALL THESE QUESTIONS CORRECTLY TO RECEIVE A BONUS!

Which action by YOU gives YOU a higher bonus?

0 cents 1 cent 2 cents 3 cents 4 cents 5 cents 6 cents 7 cents 8 cents 9 cents 10 cents

Which action by YOU gives the OTHER PLAYER a higher bonus?

0 cents 1 cent 2 cents 3 cents 4 cents 5 cents 6 cents 7 cents 8 cents 9 cents 10 cents

Which action by the OTHER PLAYER gives the OTHER PLAYER a higher payoff?

0 cents 1 cent 2 cents 3 cents 4 cents 5 cents 6 cents 7 cents 8 cents 9 cents 10 cents

Which action by the OTHER PLAYER gives YOU a higher payoff

0 cents 1 cent 2 cents 3 cents 4 cents 5 cents 6 cents 7 cents 8 cents 9 cents 10 cents

Supplementary Figure S2. PD instructions screen.

WHAT IS YOUR CHOICE?

Tick the amount of money you want to transfer to the other participant.

0 cents 1 cent 2 cents 3 cents 4 cents 5 cents 6 cents 7 cents 8 cents 9 cents 10 cents

Supplementary Figure S3. PD decision screen.

This is the second part of the HIT.

You are paired together with another, anonymous participant, different from the one with whom you were paired in the first part of the HIT. This time how much money you earn depends only on your own choice.

You are given 10 additional cents and you have to decide how much, if any, to DONATE to the other participant.

The other participant has no choice: she or he will get your donation.

WHAT IS YOUR DONATION?

Tick the amount of money you would like to donate to the other participant.

0 cents 1 cent 2 cents 3 cents 4 cents 5 cents 6 cents 7 cents 8 cents 9 cents 10 cents

Supplementary Figure S4. DG instructions and decision screen.
